# Interaction of Bioactive Mono-Terpenes with Egg Yolk on Ice Cream Physicochemical Properties

**DOI:** 10.3390/foods10081686

**Published:** 2021-07-21

**Authors:** Mostafa Gouda, Long Sheng, Rana Muhammad Aadil, Yuanyuan Liu, Meihu Ma, Xiaoli Li, Yong He, Paulo E. S. Munekata, José M. Lorenzo

**Affiliations:** 1 College of Biosystems Engineering and Food Science, Zhejiang University, 866 Yuhangtang Road, Hangzhou 310058, China; xiaolili@zju.edu.cn (X.L.); yhe@zju.edu.cn (Y.H.); 2 Department of Nutrition & Food Science, National Research Centre, Dokki, Giza 12622, Egypt; 3 National Research and Development Center for Egg Processing, College of Food Science and Technology, Huazhong Agricultural University, Wuhan 430070, China; stheaven16@126.com (L.S.); hzauliuyuan@163.com (Y.L.); 4 National Institute of Food Science and Technology, University of Agriculture, Faisalabad 38000, Pakistan; dilrana89@gmail.com; 5 Centro Tecnológico de la Carne de Galicia, Rúa Galicia No 4, Parque Tecnológico de Galicia, San Cibrao das Viñas, 32900 Ourense, Spain; jmlorenzo@ceteca.net; 6 Área de Tecnología de los Alimentos, Facultad de Ciencias de Ourense, Universidad de Vigo, 32004 Ourense, Spain

**Keywords:** bioactive molecules, egg yolk, ice cream structure, multivariate analysis models, trans-cinnamaldehyde, thymol

## Abstract

Using natural multi-function phytochemicals could be one of the best solutions for clean-label production. In this study, dairy ice creams were prepared containing 14% egg yolk and 0.1% of thymol (THY), trans-cinnamaldehyde (TC), menthol (MEN), or vanillin (VAN). Then, the physical, chemical, and structural characteristics were evaluated. Magnetic resonance imaging (MRI) analysis (a rapid, chemical-free, and non-invasive tool) was carried out to evaluate the water distribution. A multivariate analysis was conducted among all studied variables. According to the results, the overrun of the MEN ice cream was significantly increased as compared to the control sample. The density was also reduced in the MEN sample. Meanwhile, the spreadability (%) of VAN was significantly increased after 6 min as compared to the control treatment. MRI analysis revealed that water distribution was significantly changed in the THY group. The firmness and viscosity of THY samples were significantly increased (*p* < 0.05). Multivariate analysis indicated that viscosity index and consistency were the top parameters affected by THY. The authors concluded that THY and VAN are promising stabilizers for ice-cream clean production.

## 1. Introduction

Using proper stabilizers is one of the major challenges in the ice cream industry because of the difficulty to enhance the texture properties with a single stabilizer. Moreover, selecting and applying the proper amount of stabilizers in ice cream formulation depends upon the total solids and fat levels of the mass [1]. However, the use of food ingredients (such as stabilizers) is being reconsidered in order to provide final products with a clean label. The term “clean label” is defined by the International Institute of Food Technologists (IFT, Chicago, IL, U.S.) as a product that adds or mixes the fewest ingredients and makes sure that those items are recognized as healthy home ingredients by consumers [2]. Likewise, Maruyama et al. [3] stated that a “clean label” food is a food product graded as “natural” and/or “organic” (such as using natural production methods) that takes into account health-related concerns.

As a multifunctional agent, egg yolk has a significant effect on ice cream texture, i.e., enhancing water retention and viscosity that prevent ice cream separation [4]. In addition, the yolk contains several components with emulsifying properties, especially lecithin (9%) [5]. Additionally, trans-cinnamaldehyde, thymol, menthol, and vanillin could be used as natural ingredients because these compounds are generally recognized as safe materials (GRAS) by the U.S. Food and Drug Administration [6]. These ingredients are generally used in the ice cream industry for increasing sensory quality (taste, smell, and mouthfeel, for instance) [7]. In addition, a recent study proved the significant effects of these phytochemicals (e.g., THY and VAN), on the stability, size, charge, and rheological properties of emulsions [8]. These compounds improved physical and oxidative stability during storage as compared to the commercial stabilizer “Purity Gum 2000”. This effect was explained by the low molecular weight and density of these compounds that influence the properties of stabilizer ingredients in foods such as dairy and egg products [9,10].

Using multivariate analysis to solve the problems related to specialized computer software has become one of the promising approaches in agricultural product evaluation and characterization [11,12]. Additionally, the use of spectroscopic analysis such as magnetic resonance imaging (MRI) as a rapid, chemical-free, and non-invasive tool can be used to characterize and generate images of the internal water distribution state, mobility, and changes in food products by clear visual images of frozen hydrocolloid samples such as stored noodles [13]. However, more reliable information should be obtained by correlating it with chemical and chemometric analyses for verification of the bands’ relationship with the standard chemical methods, where the concentration of a complex compound is determined by the synergistic combination of multiple bands [14].

Several analyses can be used to evaluate the physical and chemical changes and problems in the ice cream industry. For instance, overrun (%) is one of the most important factors correlated with ice cream texture properties. The differences in the ice cream texture and rheological characteristics could be due to crosslink protein interactions and changes in its dairy and yolk protein structures [4,15]. Gouda et al. [12] mentioned that multivariate chemometrics models could be the best solution in solving the large amount of data generated from physicochemical methods by the synergistic combination of these different methods. As an experimental example, the use of proper stabilizers is one of the biggest problems in the ice cream industry.

This study aimed to evaluate the physical and structural effects of four safe natural alternatives to the synthetic substances representing mono-terpenes and phytochemical groups (thymol as phenol, trans-cinnamaldehyde as phenyl aldehyde, menthol as cyclic alcohol, and vanillin as phenol-aldehyde) mixed with egg yolk in producing different types of dairy ice cream products. Firmness, consistency, cohesiveness, viscosity, yield stress, microstructure by MRI, overrun, density, spreadability, and color characteristics were evaluated to provide evidence of their applicability as an alternative stabilizer in the design of ice cream formulations and consider their positive effects in the production of ice cream. In addition, a multivariate analysis of data was carried out to evaluate the physical and structural effects, providing evidence of integrating these analytical methods in the field of ice cream engineering.

## 2. Materials and Methods

### 2.1. Materials

Grade A eggs were purchased from the Huazhong Agricultural University (Wuhan, China), 3.5% fat milk was purchased from Weidendorf Ltd. (Bayern, Germany), and 35% fat whipping cream was purchased from Nestle Co. Ltd. (Frankfurt, Germany). Gelatin was purchased from Henan En Mian Food Co. Ltd. (Henan, China) and sucrose fine sugar powder was purchased from Guangdong Xiang Food Co. Ltd. (Taiwan, China). Thymol (99%; PubChem CID:6989), and vanillin (99.5%; PubChem CID:1183) were purchased from Sinopharm, Co. Ltd. (Shanghai, China), trans-cinnamaldehyde (98%; PubChem CID:637511) was purchased from Aladdin Industrial Co. Ltd. (Shanghai, China) and menthol (98%; PubChem CID:165675) was purchased from Tokyo Chemical Industry Co. Ltd. (Tokyo, Japan).

### 2.2. Sample Preparation

The eggs were manually broken, and yolks were separated from the albumen. Each yolk was carefully rolled on a filter paper (Whatman, Maidstone, UK) to remove traces of albumen that adhered to the vitelline membrane. The vitelline membrane was then disrupted with a scalpel blade, and the yolk was collected in a cooled beaker. Five egg yolk mixtures were prepared following the protocol indicated by Gouda et al. [16] as the highest concentration that has a significant impact on yolk physicochemical structure: control (egg yolk without any additive), TC (0.1% of trans-cinnamaldehyde in egg yolk), THY (0.1% of thymol in egg yolk), MEN (0.1% of menthol in egg yolk), or VAN (0.1% of vanillin in egg yolk).

For ice cream preparation, concentrations of the following materials were used: 35% milk (3.5% fat), 35% cream (35% fat), 15% sugar, 14% egg yolk mixture, and 1% gelatin [17]. Milk, cream, and gelatin were mixed and warmed up to 80 °C. Concomitantly, egg yolk mixtures were mixed with sugar using a lab mixer (MX-F, Sci-LOGEX, Beijing, China). Then, all ingredients were mixed in the mixer and pasteurized at 80 °C for 60 s, with a magnetic stirrer/heater (LL-LE10SH4C, LLES Inc., Mississauga, ON, Canada), and after that were rapidly homogenized at 12,000 rpm at 5 °C in a homogenizer (FJ-200, Shanghai Specimen and Manufactory, Shanghai, China). The mixed materials were aged overnight at 4 °C and then poured into the ice cream mixer (ICM-700, Fuxin Co., Beijing, China) for 2 h under −7 °C. Finally, the aged and processed samples from the ice cream mixer were kept at −17 °C for 24 h before the analyses.

### 2.3. Texture Properties Analysis (TPA)

An aliquot of 100 g from the control and prepared ice cream samples was put in a standard-size back extrusion container (50 mm diameter) for measuring the texture by TA-XT-Plus texture analyzer (Stable Microsystems, Surrey, UK). Compression test was carried out with a load cell of 5000 g, and 35 mm diameter cylindrical probe was used a texture profile analysis and a pre-speed of 2 mm/s, test-speed of 2 mm/s, penetration depth 15 mm, and trigger force 5.0 g to characterize 80 g of samples. Firmness, cohesiveness, consistency, and index of viscosity were calculated from the TPA software system. The average value of three replications was used for the data analysis [17].

### 2.4. Rheological Properties Analysis

Rheological measurements were carried out according to the flow behavior of the samples after 60 min at 5 °C by using a discovery hybrid rheometer (DHA-2000, TA Instruments, New Castle, DE, USA) with a parallel geometry sensor (60 mm diameter, 2.0° cone plate, Peltier plate Aluminum) and 1 mm gap following the method of Gouda et al. [10] with some modifications. In brief, the function of the shear rate from 0.1 to 100 s^−1^ was obtained for measuring viscosity and yield stress. The experimental time was adjusted to 120 s. A total of 32 points was collected with linear distribution and duplicates presented differences lower than 10%. Data obtained were adjusted to the Power law and Herschel–Bulkley models by using Trios Software Version: 3.1.0.3538.

### 2.5. Magnetic Resonance Imaging (MRI)

MRI was used to analyze the internal water distribution state, mobility, and changes of the ice cream samples by an imaging system [18]. The experiment was performed by using NMI20-015V-I equipment (Niumag Co., Shanghai, China). A good signal-to-noise ratio was achieved with four accumulations. Aliquots of 2 g from samples were placed in the instrument tube and kept at −20 °C for 16 h. After that, the ice cream sample measurements were performed directly at 0 min and the same sample was measured again after 6 min of incubation at 32 °C.

The experimental conditions were: FOV of 100 mm/100 mm, width of slice of 2.5 mm, gap of 0.5 mm, no. 4, size of reading of 256, size of phase of 192, T1 weighted image TE of 19 ms and TR of 300 ms, and T2 weighted image TE of 50 ms and TR of 1600 ms. Three layers of images of each sample are taken (high layer, middle layer, and low layer). The gradient colors from blue and red represent the highest amplitude whereas orange color represents the lowest amplitude. The surface water is characterized by higher amplitudes colors.

### 2.6. Overrun Analysis

Overrun was determined according to the method described by Adhikari et al. [19]. First, 100 mL of ice cream mixtures were weighed, and the same volume was weighed again after the ice cream-making process. Overrun (%) measurements were taken in triplicate and calculated using the Equation (1):

(1) $$\mathrm{Overrun}\left(\%\right)=\frac{\mathrm{MW}-\mathrm{IW}}{\mathrm{IW}}*100$$

where MW is the mixture weight and IW is the ice cream weight for the same sample.

### 2.7. Density Analysis

Density was determined according to the modified method described by Pourashouri et al. [20]. First, a fixed volume of ice cream mixtures was weighed, and the same volumes were weighed again after the ice cream-making process. Density measurements were taken in triplicate using the Equation (2):

(2) $$\mathrm{Density}\;\left(\frac{g}{{\mathrm{cm}}^{3}}\right)=\frac{\mathrm{Sample}\;\mathrm{weight}\;\left(g\right)}{\mathrm{Sample}\;\mathrm{volume}\;\left({\mathrm{cm}}^{3}\right)}$$

### 2.8. Spreadability Analysis

A weight of 20 g from the ice cream samples was placed separately on plastic cubes of 2 × 2 × 2 cm^3^. Then the samples were frozen at −20 °C for 12 h. Then, the plastic containers were placed in a tray along with a 30 cm rule. The ice cream cube samples were put next to a ruler with a lab glass plate and a weight of 100 g on top at 21 °C. After that, the diameter of the examined samples was taken every 2 min for 12 min, six times for each ice cream treatment [21].

### 2.9. Color Analysis

The color of the samples was expressed using the CIELAB system: L* (lightness, whiteness or brightness), a* (redness or greenness), and b* (yellowness or blueness) [16]. The measurements were performed using an automatic color chroma meter (CR-400/410, Konica Minolta Sensing Inc., Tokyo, Japan). A fixed amount, about 15 mL, of samples was poured into the measurement cell. Three replicates of each sample were measured.

### 2.10. Statistical Analysis

Data statistical analyses was performed using SPSS 16.0 (IBM, New York, NY, USA), Origin 2021 (Northampton, MA, USA), MetaboAnalyst software (MetaboAnalystR, Edmonton, AB, Canada), and Matlab R2017b (MathWorks, Natick, MA, USA). Principal component analysis (PCA) and other multivariate discriminant analyses (DA) were analyzed using the Partial Least Squares (PLS) model. Clustering of treatments was assessed with PCA score plots and heatmeat projections. The relevance of each analysis in the experimental data variance was evaluated using the variable importance in the projection (VIP). The different colored boxes in the heatmap and the VIP represent significant differences (*p*-value ≤ 0.05) [11]. Analysis of variance (ANOVA) was applied with *p*-value < 0.05. The least significant differences (LSD) and Duncan tests were used to determine significant differences among the tested samples, in which, mean ± SD with different Roman alphabet superscripts indicates that the values differ significantly (*p*-value ≤ 0.05) and (a < b < c < d …). In addition, * and ** asterisks were used as the column differs significantly (*p* ≤ 0.05) and (*p* ≤ 0.01), respectively). The Pearson correlation coefficients (r^2^) for the relationships between the properties were also calculated.

## 3. Results and Discussion

### 3.1. Texture Properties

**Figure 1:**
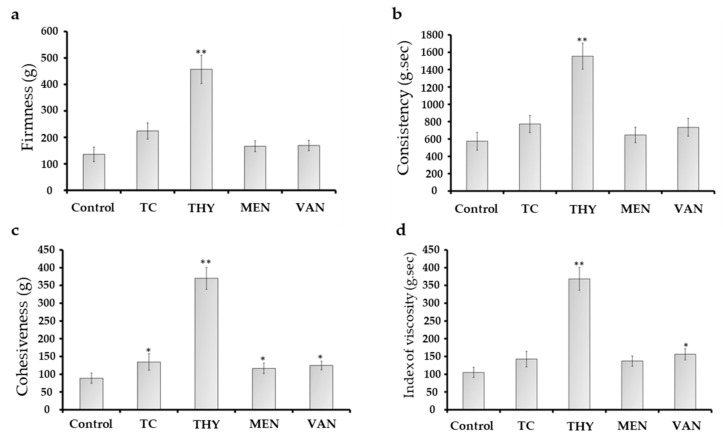
Texture properties of the different mixtures of TC, THY, MEN, or VAN egg yolk ice creams. (**a**) Firmness, (**b**) Consistency, (**c**) Cohesiveness, (**d**) Index of viscosity. *, **: The column differs significantly ((*p* ≤ 0.05) and (*p* ≤ 0.01), respectively).

Ice cream texture properties are the most important parameters in the dairy industry [22]. The effect of egg mixtures in ice cream texture values is shown in Figure 1. In the THY group, firmness value (457.00 ± 53.56 g) was significantly increased (*p* < 0.05) as compared to the control (135.85 ± 27.61 g). El-Sayed et al. [9] mentioned that the significant enhancement in dairy ice cream texture properties is due to the interactions between the phenol ring and ice cream proteins. The potential mechanism could be from the complex structure of these molecules’ rings (such as THY phenolic ring) with yolk phospholipids and milk casein protein cross-links that mainly improve their hydrophobic interactions and the physicochemical characteristics such as texture and its stability as mentioned by Gabbi et al. [23].

THY treatment showed the highest values (*p* < 0.05) in the cohesiveness (369.69 ± 30.64 g) (Figure 1c) and consistency (1553.58 ± 150.75 g·s; Figure 1b), as compared to the control with 88.56 ± 14.38 g and 573.92 ± 102.46 g·s, respectively. The same behavior was observed in firmness and Index of viscosity (Figure 1a,d).

The increase in THY and VAN cohesiveness and consistency might be due to the changes in the hydrophobic core of yolk granular molecules in the ice cream. Moreover, the increase in texture levels of ice cream is generally related to the reduction in the growth rate of ice crystals in the ice cream [19]. Jardines et al. [22] reported that thermal properties (such as melting rate and spreadability) are correlated with textural properties wherein sample resistance to the compression process is proportional to ice cream temperature stability.

### 3.2. Rheological Properties

The effects of TC, THY, MEN, or VAN on the ice cream rheological properties such as viscosity and stress yield are shown in Table 1. All the presented samples have shown non-Newtonian behavior in the studied range of shear rate. Therefore, two models of calculations, the Power-law model and Herschel–Bulkley model, were used to calculate the viscosity and rate index.

According to the Power-law model, the viscosity of the THY group was significantly (*p* < 0.05) increased (27.92 ± 5.25 Pa·s) as compared to the control (21.24 ± 0.15 Pa·s). Conversely, the viscosity of the MEN group was significantly decreased to 15.68 ± 1.24 Pa·s in relation to the control sample. Moreover, a high positive correlation (r^2^ = 0.90) was found between the Index of viscosity and cohesiveness.

According to the Herschel–Bulkley model, the yield stress of the THY group was significantly (*p* < 0.05) increased (30.88 ± 5.80 Pa·s) as compared to the control (20.47 ± 6.08 Pa·s). Differently, the viscosity of the MEN group was significantly decreased (17.46 ± 1.37 Pa·s). Additionally, a positive correlation (r^2^ = 0.77) was found between stress yield and consistency. Anjo et al. [24] reported that adding additives to ice cream products that can change the gel-like protein networks would modify ice cream rheology and pseudo-plasticity. Consequently, a positive impact on its texture properties could be observed. Moreover, in our recent studies, THY and VAN showed a significant improvement in the physicochemical structure of biological media due to changes in the secondary protein structure, consequently changing the media functionality [12,16]. In addition, Jardines et al. [22] reported that the increase in apparent viscosity could be explained by the interactions of sugars and the liquid components of the ice cream mix. This effect is caused by both the contribution of soluble solids to the aqueous phase and by enhancing the water-binding ability of the macromolecules such as the available proteins and carbohydrates, which form a gel-like structure that modifies the viscosity mix and the ice cream stability. Such rheological properties are also affected by the formed ice crystals size and distribution, and the extent of fat deterioration.

### 3.3. Magnetic Resonance Imaging (MRI)

**Figure 2:**
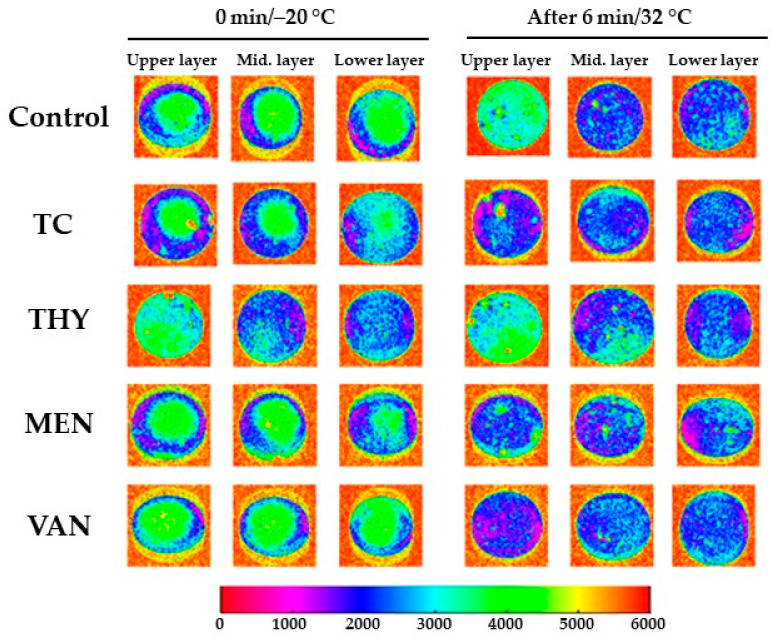
MRI images of TC, THY, MEN, or VAN egg yolk ice creams at 0 min/−20 °C and after 6 min/32 °C.

The visualization of MRI images is presented in Figure 2. In the TC freezing state (0 min), it was observed that the thickness of the outside sphere (representing the distribution of the water phase) was larger than observed for the control samples (Figure 2). However, after 6 min of incubation at 32 °C, the distribution of oil and water changed, and some low-density compounds remained in the middle and lower layers. Particularly for the THY group in the freezing state, the distribution of oil and water changed in comparison to the control. In addition, it was found that water was in the center of the middle and lower layers. However, a low water content was observed in the upper layer of the THY sample. After 6 min of incubation at 32 °C, the distribution of oil and water did not change, and this sample was more stable than the control and the TC groups. Additionally, some low-density compounds remained in the middle and lower layers as a good indication of the sample’s stability, which supports the stability of the ice cream treatment under 32 °C.

This behavior can be explained by the ability of thymol to enhance the emulsification capacity of egg yolk. This effect was attributed to the hydrophobic core changes in ice cream macromolecules. Therefore, the increasing viscosity effect of THY group was more consistent and produced thick non-absorbing layers, which can be justified by the rheology and texture measurements. Develioglu et al. [25] reported that a high intrinsic viscosity leads to a high hydrodynamic volume, which increases the stability of ice cream. Furthermore, Cheng et al. [26] mentioned that MRI is an effective tool to study the real-time fluid distribution in the natural core. It can explain the changes in the ice cream layers’ physical characteristics during the melting process, resulting in different physicochemical discrimination among ice cream samples.

### 3.4. Overrun Evaluation

**Figure 3:**
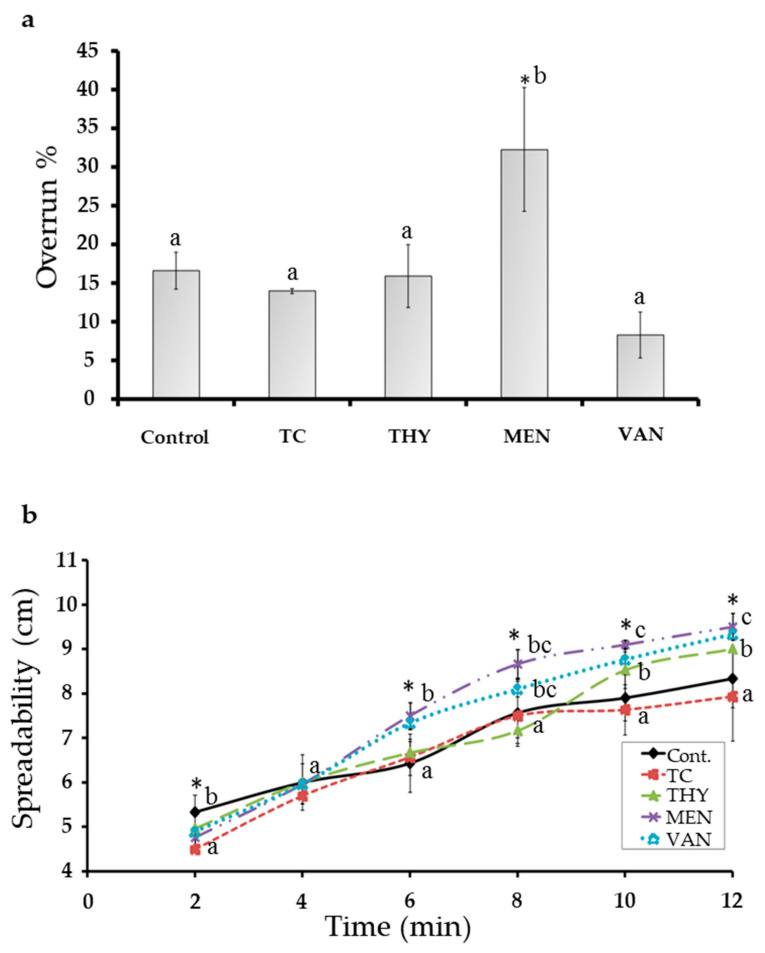
(**a**) Overrun % of the different mixtures of TC, THY, MEN, or VAN egg yolk ice creams; (**b**), Spreadability patterns of the different studied groups. Mean ± SD with different Roman alphabet and asterisk (*) indicate that the values differ significantly (*p*-value ≤ 0.05; a < b < c) at the different columns and the same time (min).

The overrun represents the air content in ice cream, which is an important indicator that affects the melting behavior, structure, texture, and sensory properties of ice cream [27]. The results of TC, THY, MEN, or VAN treatments on the ice cream overrun are presented in Figure 3a. Only MEN overrun value (32.25 ± 7.98%) was significantly (*p* < 0.05) higher than observed for the control sample (16.61 ± 2.39%). The authors of [28] proved that the high viscosity of ice cream mixtures may prevent a strong shakeup and air incorporation, which prevents the increase in the air contents and overrun values. Therefore, the high viscosity and texture of THY and VAN treatment (Table 1 and Figure 1a) might have limited the formation of air bubbles, which affected the overrun [29]. In line with this outcome, Yan et al. [27] mentioned that the viscosity rate has a noticeable effect on the overrun values. However, in the case that the film rapidly drains among air bubbles, the air bubbles coalesce [14], which may be the reason for the highest overrun value obtained in the MEN group.

**Figure 4:**
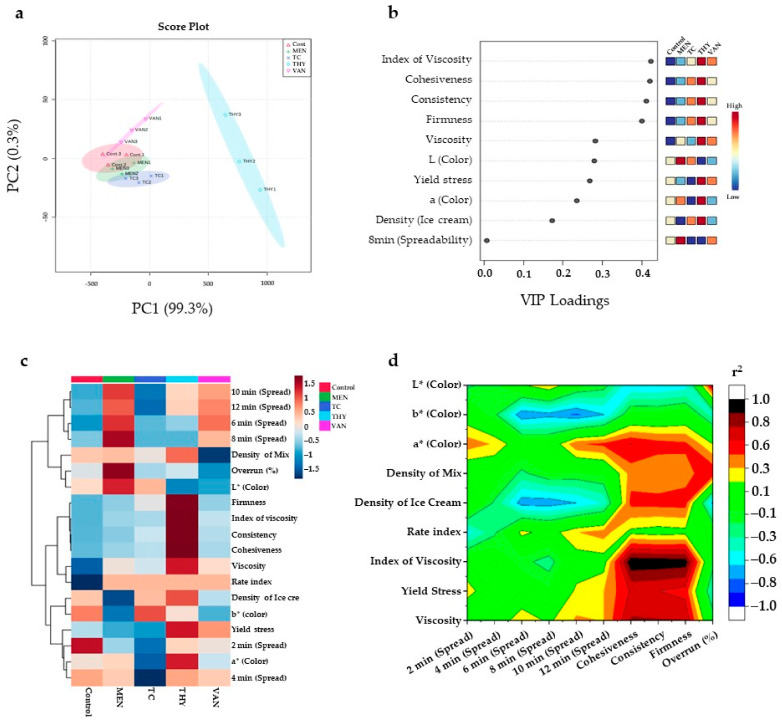
Multivariate analysis, and correlations of TC, THY, MEN, or VAN egg yolk ice cream physicochemical analyses; (**a**) Principle component analysis (PCA) scores; (**b**) Variable importance in projection (VIP) classification by PLS-DA for the entire analyses; (**c**) Clustering results shown as heatmap; the colored boxes on the right indicate the relative intensity for the species; (**d**) Correlation (r^2^) summarization between the full the physicochemical parameters.

A negative correlation (r^2^ = −0.65) was found between stress yield and overrun (Figure 4d). Similarly, Adhikari et al. [19] mentioned that the overrun is negatively correlated with ice cream texture. This could be due to the presence of the larger volume of a compressible dispersed part (e.g., the large ice crystals), which leads to less resistance to an applied force.

### 3.5. Density Results

The density results of mixtures and ice cream samples are shown in Table 2. It was observed that density decreased after the ice cream formation stage. For the density of the mixtures, VAN group density was significantly (*p* < 0.05) decreased (0.84 ± 0.03 g/cm^3^) as compared to the control (0.94 ± 0.01 g/cm^3^). However, the density of the ice cream in the MEN group was significantly decreased to 0.71 ± 0.00 g/cm^3^ as compared to the control (0.81 ± 0.02 g/cm^3^). Ismail et al. [30] mentioned that ice cream density is affected by the incorporation of air in the ice cream mixture during the pre-freezing process, which decreases the specific gravity of the resultant ice cream. This may be due to the specific gravity of formula components in addition to the mixture’s ability to hold the air bubbles. Therefore, the density is significantly correlated with the viscosity and significantly affects the air content of ice cream products [20].

### 3.6. Spreadability Results

As seen in Figure 3b, the spreadability of the TC, THY, MEN and VAN groups was significantly different (*p* < 0.05) from the control. After 2 min, the control group was significantly higher (*p* < 0.05) than other groups. While after 4 min, all five groups had no significant differences among them. After 6 min, significant differences among groups were observed, but the THY and TC groups had similar spreadability in comparison to the control ice cream.

The spreadability of the MEN and VAN groups was significantly (*p* < 0.05) higher than that observed in the control sample until 12 min. These results are negatively attributed (r^2^ = −0.80) to the results of ice cream density (Figure 4d). Therefore, the increase in the ice cream density decreased the spreadability of the ice cream. These observations are in agreement with Fiol et al. [21], who reported that spreadability is correlated with the overall stability of the ice cream.

### 3.7. Color Properties

The variation in color parameters, lightness (L*), redness (a*) and yellowness (b*) is shown in Table 3. The addition of menthol and trans-cinnamaldehyde significantly (*p* < 0.05) increased the lightness of the ice cream in comparison to the control samples. Regarding the yellowness, the b* values for the MEN and VAN treatments were significantly lower than those obtained from the control (22.46 ± 0.76, 23.18 ± 0.59, and 25.44 ± 0.48, respectively). One possible explanation for changes in the color parameters (especially for yellowness) is due to the fact that surface hydrophobicity changes with the amount of fat in the surface, which has the majority of yolk hydrophobic pigments in the ice cream [16,31].

### 3.8. Multivariate Analysis

The principal component analysis (PCA) among all studied groups is presented in Figure 4a, and the differences among groups were evaluated according to the contribution rate of PC factors [6,32]. THY and TC were significantly (*p* < 0.05) different from the control. Meanwhile, from VIP and heatmap (Figure 4b,c), it was found that viscosity index, cohesiveness, and consistency were the top affected parameters with the highest influences on the THY group as compared to the control. Also, the VIP multivariate model indicated that THY and VAN significantly (*p* < 0.05) increased the cohesiveness and consistency of ice cream (Figure 4b). This outcome can be attributed to the significant effect of mono-terpenes in THY group in changing the secondary protein structure and the oil-to-water distribution in the biological media [12].

Figure 4d represents the correlations (r^2^) heatmap among all studied parameters. A positive correlation (r^2^ = 0.75) was found between L* value and overrun. The b* value was negatively correlated (r^2^ = −0.93) with spreadability after 8 min to 12 min (Figure 4d). Moreover, a high positive correlation (r^2^ = 0.90) was found between the Index of viscosity and cohesiveness. Our results are in agreement with Harlina et al. [33], who found a significant influence of eugenol (terpene) on egg PCA due to its effect on lipid construction and the enzymatic pathways and the lipoxygenases. Additionally, our results confirm that the granular protein structure has an effect on system behavior. Therefore, THY significantly increased the share rate and texture of ice cream. This effect can be explained by the changes in the diameter size and the hydrophobic core of yolk granular molecules in the ice cream, which negatively affected lightness [34].

## 4. Conclusions

In this study, four bioactive compounds with egg yolk (TC, THY, MEN, and VAN) were evaluated for obtaining a high-quality ice cream product. These four compounds showed different effects on the functional properties of ice creams. Different significant responses were observed in the following analyses (overrun, density, firmness, consistency, cohesiveness, viscosity, yield stress, color, spreadability, and microstructure influences by MRI). Therefore, it can be concluded that natural phytochemicals (especially THY and VAN) can play an important role with egg yolk as a natural stabilizer in the design of ice cream formulations. The advances in the pretreatment with bioactive terpenes for ice cream production came from the changes in the yolk functionality, which can direct the production without any further chemical or physical assistance. This study can support the applicability of natural bioactive molecules as a simple “clean label” strategy. Furthermore, large-scale studies are needed to optimize the used formulations together with the other processing conditions in view of their industrial applications.

## Figures and Tables

**Table 1:** Rheological results of TC, THY, MEN or VAN egg yolk ice creams in mixtures and after ice-cream process.

	Power Law Model		Herschel-Bulkley Model		
Treatments	Viscosity (Pa·s)	Rate Index	Yield Stress (Pa)	Viscosity (Pa·s)	Rate Index
Control	21.24 ± 0.15 ^b^	0.22 ± 0.04 ^a^	20.47 ± 6.08 ^ab^	1.27 ± 0.18 ^a^	0.76 ± 0.25 ^a^
TC	14.86 ± 0.93 ^a^	0.31 ± 0.00 ^b^	16.56 ± 1.03 ^a^	1.76 ± 0.08 ^b^	0.90 ± 0.00 ^a^
THY	27.92 ± 5.25 ^c^	0.24 ± 0.00 ^a^	30.88 ± 5.80 ^c^	2.36 ± 0.44 ^c^	0.90 ± 0.00 ^a^
MEN	15.68 ± 1.24 ^a^	0.30 ± 0.01 ^b^	17.46 ± 1.37 ^a^	1.84 ± 0.00 ^b^	0.90 ± 0.00 ^a^
VAN	24.10 ± 3.90 ^bc^	0.23 ± 0.02 ^a^	26.59 ± 4.17 ^bc^	1.89 ± 0.05 ^b^	0.90 ± 0.00 ^a^

Mean ± SD with different Roman superscripts within the same column indicate that the values differ significantly (*p* ≤ 0.05).

**Table 2:** Density results of TC, THY, MEN or VAN egg yolk ice creams in mixtures and after ice cream process.

Groups	Mixture Density (g/cm^3^)	Ice Cream Density (g/cm^3^)
Control	0.94 ± 0.01 ^b^	0.81 ± 0.02 ^b^
TC	0.92 ± 0.01 ^b^	0.81 ± 0.01 ^b^
THY	0.97 ± 0.01 ^b^	0.84 ± 0.04 ^c^
MEN	0.94 ± 0.06 ^b^	0.71 ± 0.00 ^a^
VAN	0.84 ± 0.03 ^a^	0.77 ± 0.01 ^b^

Mean ± SD with different Roman superscripts within the same column indicate that the values differ significantly (*p* ≤ 0.05).

**Table 3:** Color results of TC, THY, MEN or VAN egg yolk ice creams in mixtures and after ice cream process.

Treatments	L*	a*	b*	ΔL*	Δa*	Δb*
Control	80.24 ± 0.06 ^b^	1.90 ± 0.05 ^b^	25.44 ± 0.48 ^cd^	54.84 ± 0.06 ^c^	1.60 ± 0.05 ^b^	23.25 ± 0.54 ^cd^
TC	80.40 ± 0.12 ^c^	1.56 ± 0.06 ^a^	25.83 ± 0.69 ^d^	54.99 ± 0.12 ^d^	1.26 ± 0.06 ^a^	23.68 ± 0.69 ^d^
THY	79.50 ± 0.03 ^a^	2.20 ± 0.10 ^c^	24.40 ± 0.85 ^bc^	54.10 ± 0.03 ^a^	1.90 ± 0.10 ^b^	22.26 ± 0.84 ^bc^
MEN	80.88 ± 0.10 ^d^	1.95 ± 0.08 ^b^	22.46 ± 0.76 ^a^	55.47 ± 0.10 ^e^	1.65 ± 0.08 ^b^	20.31 ± 0.76 ^ab^
VAN	79.65 ± 0.08 ^a^	1.84 ± 0.06 ^b^	23.18 ± 0.59 ^ab^	54.25 ± 0.08 ^b^	1.54 ± 0.06 ^b^	21.03 ± 0.58 ^a^

Mean ± SD with different Roman superscripts within the same column indicate that the values differ significantly (*p* ≤ 0.05).
